# A workflow for neutron activation analysis of archaeological ceramics at the Atominstitut in Vienna, Austria

**DOI:** 10.1007/s10967-018-5803-7

**Published:** 2018-03-14

**Authors:** Johannes H. Sterba

**Affiliations:** Atominstitut, TU Wien, Stadionallee 2, 1020 Vienna, Austria

**Keywords:** Neutron activation analysis, Archaeological ceramics, Archaeometry

## Abstract

The main focus for neutron activation analysis (NAA) at the Atominstitut in Vienna has moved to the analysis of archaeological ceramics. The workflow for NAA has been adapted for this material and the elemental spectrum quantified has been expanded for compatibility with international databases. Statistical methods for the grouping of the archaeometric data have been implemented, following the methods applied by Mommsen et al. in Bonn (Archaeometry 30(1):47–57, 1988). Limits of detection specific for ceramics have been calculated and are at the ng/g level. High reproducibility as necessary for archaeometric analysis can be shown by comparative measurements of an internal quality control sample.

## Introduction

The Atominstitut of the TU Wien in Vienna, Austria operates the only research reactor in Austria. The TRIGA Mk II reactor operates at 250 kW thermal power in a steady state and can be pulsed to 250 MW. First criticality was in 1962 and from the very start, Neutron Activation Analysis was part of its applications.

Members of the Atominstitut contributed to Neutron Activation Analysis in a methodological way [1–5], most famous for the invention of Loss Free Counting [6, 7]. But in parallel to the methodological development, applications of the methods to many different materials and research questions was also done [8–13]. With the beginning of the special research project SCIEM2000 [14, 15], Neutron Activation Analysis was applied to archaeological material. At first, the main focus was the geochemical characterization of volcanic material such as pumice or obsidian [16–20] but in 2009, ceramics became the focus of Neutron Activation Analysis at the Atominstitut [21].

The methods used for silicate rock samples [22] were modified and the elements measured extended to better fit the different material but also to be able to apply the statistical methods in use in Bonn [23, 24].

This work describes the routine procedures used to sample, analyse and statistically evaluate ceramic material at the Atominstitut.

## Methods

### Sampling

For sampling, two alternative methods are used. The preferred sampling procedure is to carefully break off a small piece of the sherd. The necessary size depends on the homogeneity of the sherd but in most cases, a piece of 10 × 10 mm is sufficient. Naturally, care is taken to not harm the typology of the sample, so any breaks are discussed before with the corresponding archaeologist.

The separate piece is then cleaned of any surface contamination or paint by scraping with a sharp silicon knife made from a single crystal. After cleaning, the sample is crushed in an agate (almost pure SiO_2_ with Mohs scale hardness of 7) mortar and ground to a homogeneous powder with an approximate mean grain size of 5 µm.

**Fig. 1:**
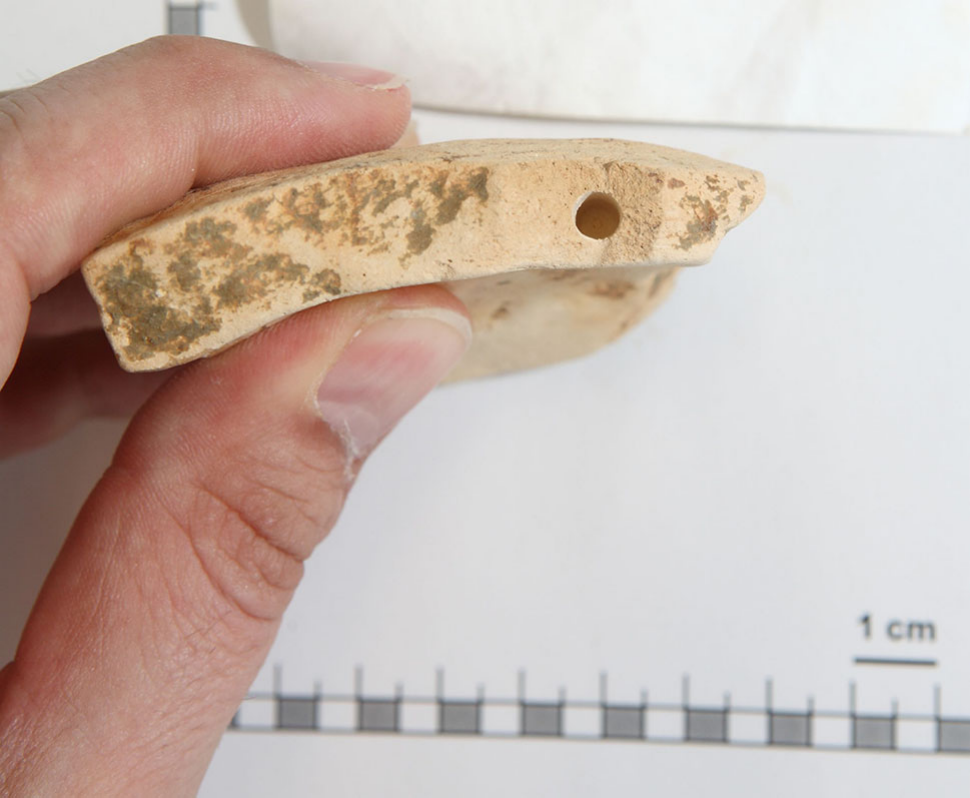
A ceramic sherd after drilling. The visible hole is approximately 3 mm in diameter and 2 cm deep

If breaking off is considered too damaging to the original sherd, an alumina drill is used to collect sample material. After consultation with the corresponding archaeologist, one or two areas where drilling is considered least harmful are identified. A hole of approximately 3 mm diameter and a depth of 2 cm is usually sufficient to provide the needed sampling material (see Fig. 1). When drilling, surface cleaning is not necessary, instead, the first half millimeter of drill cuttings is discarded.

The drill cuttings collected from drilling are very similar to the material collected by crushing and grinding as described above. The resulting powder is then dried over night in an oven at 100 °C to remove humidity from the sample. After drying, between 100 and 150 mg of the homogenized sample material are weighed into Suprasil™ quartz glass vials which are then sealed.

Where sampling in the field is required, both methods have been applied successfully. In case of drilling, only the drill bits have to be transported since they are fitted to be used with commercially available (cordless) electric screwdrivers. The material obtained by drilling can then easily be transported or mailed. In cases were the breaking off of small pieces is permitted in the field, sherds are collected and packed in PE bags for transport. All further cleaning and crushing is then done in the laboratory.

### Irradiation and measurement

For a measurement run, up to 43 samples together with 5 international certified reference materials (CANMET reference soil SO1, NIST SRM 1633b Coal Fly Ash, Light Sandy Soil BCR No. 142, NIST SRM 2702 Inorganics in Marine Sediment, and MC Rhyolite GBW 07113), the reference material used in Bonn [25] for database compatibility as well as an internal quality control sample [26] are irradiated together in an irradiation capsule at the central irradiation position in the TRIGA Mk II reactor and an approximate neutron flux density of 1 × 10^13^ cm^−2^ s^−1^ for 35–40 h. After irradiation and a decay time of 4 days, the irradiation capsule is recovered, the individual vials’ surface is decontaminated and the vials are packed into PE containers that fit the sample changer of the gamma spectroscopy system at the Atominstitut. Samples are sequentially measured in a first measurement for 1800 s each and, after an additional decay time of 3 weeks, for 10,000 s each. For gamma spectroscopy, a 222 cm^3^ HPGe-detector (1.78 keV resolution at the 1332 keV ^60^Co peak; 48.2% relative efficiency), connected to a PC based multi-channel analyzer with digital preloaded filter and loss free counting system, is used.

Quantitative Analysis is performed on the gamma spectra by comparison to the certified values of the reference materials. Elemental concentrations for the following elements are collected: Na, K, Sc, Cr, Fe, Co, Ni, Zn, As, Rb, Sr, Zr, Sb, Cs, Ba, La, Ce, Nd, Sm, Eu, Tb, Yb, Lu, Hf, Ta, W, Th, U. Selection of elements follows both geological significance as well as convenience. Experience shows that additional elements which would only be measureable by performing an additional (short time) irradiation and subsequent measurement like Al, Mg, Cl, Mn, V, Dy etc. do not contribute significantly to the chemical fingerprint but would severely decrease sample throughput. Table 1 details which nuclear reactions and isotopes are used for each element, as well as which measurement (first or second) is used for evaluation.

**Table 1:** Nuclear reactions and gamma energy peaks used for the qualification and quantification of elements as well as the respective limit of detection

Element	Reaction	Energy (keV)	Measurement	Detection limit (ng/g)	Half-life
Na	^23^Na(n,$\gamma$)^24^Na	2754	1	310	14.997 h
K	^41^K(n,$\gamma$)^42^K	1525	1	17,000	12.355 h
Sc	^45^Sc(n,$\gamma$)^46^Sc	1120.5	2	0.10	83.79 day
Cr	^50^Cr(n,$\gamma$)^51^Cr	320.1	2	16	27.704 day
Fe	^58^Fe(n,$\gamma$)^59^Fe	1099.2	2	800	44.495 day
Co	^59^Co(n,$\gamma$)^60^Co	1173.2	2	1.3	1925.28 day
Ni	^58^Ni(n,p)^58^Co	810	2	43	70.86 day
Zn	^64^Zn(n,$\gamma$)^65^Zn	1115.5	2	32	243.93 day
As	^75^As(n,$\gamma$)^76^As	559.1	1	2.8	26.24 h
Rb	^85^Rb(n,$\gamma$)^86^Rb	1077	2	29	18.642 day
Sr	^84^Sr(n,$\gamma$)^85^Sr	514	2	260	64.849 day
Zr	^94^Zr(n,$\gamma$)^95^Zr	756.7	2	250	64.032 day
Sb	^123^Sb(n,$\gamma$)^124^Sb	1691	2	1.2	60.20 day
Cs	^133^Cs(n,$\gamma$)^134^Cs	795.9	2	0.72	2.0652 a
Ba	^130^Ba(n,$\gamma$)^131^Ba	496.3	2	340	11.50 day
La	^139^La(n,$\gamma$)^140^La	1596	1	3.2	1.67855 day
Ce	^140^Ce(n,$\gamma$)^141^Ce	145.4	2	13	32.511 day
Nd	^146^Nd(n,$\gamma$)^147^Nd	531	2	100	10.98 day
Sm	^152^Sm(n,$\gamma$)^153^Sm	103.2	1	0.39	46.284 h
Eu	^151^Eu(n,$\gamma$)^152^Eu	1408	2	0.41	13.517 a
Tb	^159^Tb(n,$\gamma$)^160^Tb	879.4	2	0.40	72.3 day
Yb	^168^Yb(n,$\gamma$)^169^Yb	177.2	2	3.0	32.018 day
Lu	^176^Lu(n,$\gamma$)^177^Lu	208.4	1	0.29	6.647 day
Hf	^180^Hf(n,$\gamma$)^181^Hf	482.2	2	2.2	42.39 day
Ta	^181^Ta(n,$\gamma$)^182^Ta	1221.3	2	0.55	114.74 day
W	^186^W(n,$\gamma$)^187^W	685.7	1	4.0	24.000 h
Th	^232^Th(n,$\gamma$)^233^Th $\to$ ^233^Pa	312.2	2	1.6	26.975 day
U	^238^U(n,$\gamma$)^239^U $\to$ ^239^Np	277.4	1	2.4	2.356 day

Peak search and spectrum evaluation is done in GENIE2000™ software. For some elements, manual checking of the spectra is necessary to resolve overlapping peak areas. This is done for the elements Sm, Lu and U routinely for all samples by manually adjusting the peak search algorithm. Furthermore, peak area values and errors are checked manually if extreme values are reached. For potential interferences from fission products, uranium content is too low (less than 5 µg/g) in most cases to contribute significantly to the comparatively large content of Zr (above 50 µg/g). Other interferences from different nuclear reactions have also been checked and found negligible for the specific case of ceramics.

### Statistical analysis

After measurement, the collected data are then further analyzed using a statistical filter method developed in Bonn in the 1970s [23, 24]. For this, the statistical calculations described by Beier and Mommsen [24] have been implemented in the statistical software R [27]. Care was taken to produce identical results to the software used in Bonn (Mommsen, personal communication). Using the filtering method, samples are iteratively grouped according to their modified Mahalanobis distance [24, 28]. This is done by (1) using the measurement errors as scaling factors for the elemental concentrations and (2) calculating the best relative fit factor for two samples [25], the so-called dilution correction. From this, the modified Mahalanobis distance between two samples can be calculated. In most cases, samples with a Mahalanobis distance of less than 2 are considered to be from the same group. After a first grouping, the mean concentrations for a group can be calculated and subsequently be compared to other samples by again applying the same algorithm. In this way, iterative grouping is achieved. For the calculation, only a subset of the measured elements is used, as is done in Bonn. The elements not used for the calculation either show a very large natural spread in ceramics (Na, As, Sr, Ba) or are usually measured with very large measurement errors (Nd, W). Both would influence the grouping in a way that pushes unrelated samples closer together due to the larger spread introduced to the dataset. For all non-grouping purposes (mean values of groups, other statistical methods, etc.), all measured elements are used.

## Experimental

Following the procedures described above and including an empty vial of Suprasil™ glass in a measurement run, it is possible to use the measurement of this sample to calculate detection limits for all elements quantified. Detection limits were calculated by using the background values measured in the empty vial following the procedures and equations described by Currie [29] and are shown in the last column of Table 1. To determine background values, peak regions of interest (ROI) from the spectra of a ceramic sample as found by the peak search algorithm were copied to the spectra of the empty glass vial. Total areas found at the determined ROIs were used for the calculation of the limits of detection using the “working” expression of 3.29 *σ*_B_ (Table 1 in [29]). This leads to lowest possible limits of detection, only possible because a well-known blank is available. Following Révay [30], limits of detection would be somewhat higher but more dependent on the specific ceramics analyzed.

For the application of the statistical filters, which use measurement errors as scaling factors, it is important to implement reliable error calculation. Measurement error of the concentration values consists of two main components: The error resulting from counting statistics and the error resulting from the used reference materials. For this second source of error, the standard error of the mean of the five reference materials is used. Errors of the certified values are not used in the calculation since, by the use of five distinctly different reference materials, the standard error of their mean arguably includes any spread contributed by deviations from the certified values. In the old workflow, only the error resulting from counting statistics was used.

Using standard error propagation, the total measurement error is calculated from the two errors (counting statistics and reference materials’ error). In regular cases, measurement error is below 10% for Nd and W, below 5% for As, Sr, Zr, Sb, Ba, Yb and Lu, and below 3% for all other elements. Naturally, in cases where measured values are close to the detection limit, measurement errors will be higher.

To identify any potential changes to the repeatability and reproducibility of the workflow established for ceramic analysis in comparison to the parameters originally used for the analysis of geological material, measurement data of the internal quality control sample “SAT 5” [23, 26] was compared between the different workflows. The “SAT 5” quality control material is a volcanic material (pumice) collected in 1998 in Santorin. Its use as internal quality control material is due to its easy availability, high homogeneity and special interest during the SCIEM2000 project.

Since 1999, the “SAT 5” material has been measured repeatedly in almost all NAA runs at the Atominstitut. Since 2009, the workflow was changed as described above. The original mean concentration as published [26] was slightly adjusted by taking into account the repeat measurements in the years following. The many measurements also lead to a good understanding of the average measurement error and the natural inhomogeneity of the material. The original (for reference) and adjusted values (see above), as well as the mean of the measurement errors and total standard deviation are presented in Table 2. It is important to note however, that in the old workflow, measurement error was only calculated from counting statistics, ignoring an additional error introduced by averaging the reference materials (see above). The difference between the measurement error and the standard deviation can be interpreted as the natural variation within the sample.

**Table 2:** Comparison of the original published data [26] of the “SAT 5” material with the mean from all measurements using the old workflow as well as the same for the new workflow

	Original data	Old workflow			New workflow		
		Mean	Average measurement error	Standard deviation	Mean	Average measurement error	Standard deviation
Na	32,200	33,700	66	1600	34,800	630	1400
K	23,500	24,100	1000	1000	25,700	6100	1100
Sc	8.75	9.05	0.0089	1.0	8.45	0.20	0.33
Cr	2.15	2.5	0.26	1.3	2.36	0.19	0.34
Fe	22,700	22,900	42	2400	21,400	370	77
Co	4.38	4.50	0.022	1.2	3.89	0.086	0.13
Ni	NA	NA	NA	NA	BDL	NA	NA
Zn	NA	70.1	1.6	9.9	54.9	1.3	2.1
As	2.60	2.84	0.37	0.75	2.78	0.15	0.26
Rb	105	105	1.3	6.5	104	2.8	3.0
Sr	NA	NA	NA	NA	78	13	22
Zr	283	285	11	17	252	18	26
Sb	0.280	0.295	0.015	0.040	0.258	0.022	0.020
Cs	2.74	2.81	0.04	0.19	2.83	0.066	0.072
Ba	552	553	12	24	529	23	16
La	31.3	31.1	0.094	1.4	30	0.79	1.0
Ce	62.3	61.2	0.29	3.5	59.5	1.5	2.4
Nd	29.0	25.1	0.91	2.5	24.9	5.4	3.7
Sm	6.02	6.03	0.012	0.28	5.61	0.16	0.33
Eu	0.973	1.00	0.0071	0.036	0.974	0.027	0.040
Tb	NA	1.01	NA	NA	0.969	0.036	0.044
Yb	5.48	4.95	0.035	0.28	4.75	0.13	0.16
Lu	0.810	0.808	0.0038	0.049	0.697	0.058	0.068
Hf	7.57	7.53	0.032	0.50	7.52	0.21	0.31
Ta	0.760	0.788	0.022	0.035	0.8	0.021	0.020
W	NA	NA	NA	NA	1.4	1	0.4
Th	19.7	19.3	0.039	1.2	18.7	0.37	0.46
U	5.89	5.69	0.15	0.39	5.47	0.34	0.45

For both workflows, the mean of the measurement errors (average measurement error) and standard deviation of all measurements is shown. In the old workflow, measurement error only included counting statistics. All values are in µg/g, NA depicts values that were not measured, BDL indicates a value below the limit of detection

**Fig. 2:**
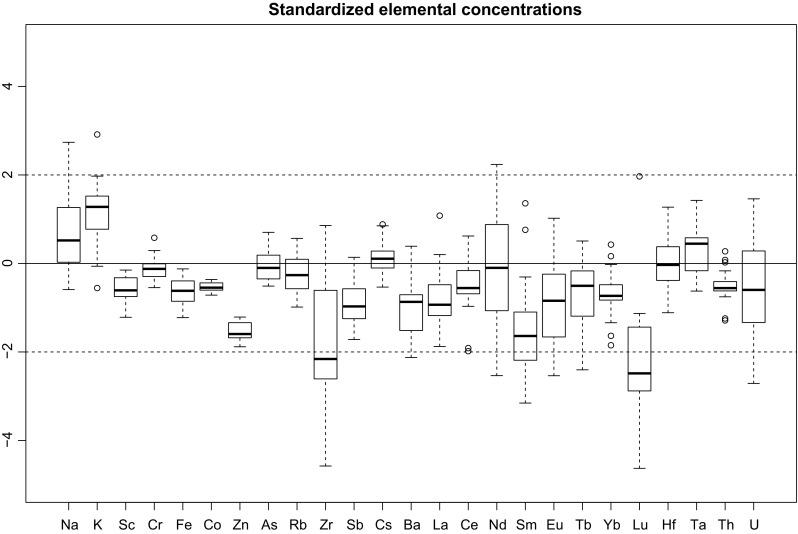
Boxplot of standardized elemental concentrations of the “SAT 5” material measured with the new workflow. Standardization follows the equation $z = (x - \mu )/\sigma$, where *µ* and *σ* are derived from the data measured using the old workflow

Using the mean value and the total standard deviation of all measurements done with the old workflow, all measurements of the “SAT 5” material done with the new workflow can be standardized to an expected mean of 0 and an expected standard deviation of 1 by the well known equation $z = (x - \mu )/\sigma$. Figure 2 shows the standardized values of all measurements of the “SAT 5” material using the new workflow in a boxplot. It can easily be seen that all measured values, with the exception of Zr and Lu fall between two standard deviations. This shows that the reproducibility of the new workflow is good. Comparison of the mean of the measurement errors with the total standard deviation (see Table 2) also shows that the measurement errors are larger than for the old workflow as a result of the inclusion of the additional error introduced by averaging over several reference materials. In general, the measurement error is still smaller than the total standard deviation of the measurements, indicating that the natural inhomogeneity of the sample is larger than the measurement error.

The deviation of Zr and Lu are due to adjustments in the evaluation of the spectra. In case of Lu, in the new workflow, the peak at 208.4 keV from the first (short) measurement is used after manual adjustment of the peak area to correct for an interfering peak at 209.5 keV. In the old workflow, the same peak, but from the second measurement has been used but without any correction. The lower values from the new workflow thus are deemed correct. In the case of Zr, in the old workflow, a non-certified value for the Zr content of the reference material CFA was used, in the new workflow it was decided to drop that value.

For the elements Na, Nd and U, the spread in Fig. 2 is large. In the case of Na and U, this is due to the fairly large natural variation, in case of Nd, the measurement error is comparatively large due to the timing of the measurements. To decrease the measurement error of Zr, an additional measurement with different decay times would be necessary. Since the error is acceptable as it is, it was decided that an additional measurement for a single element was not productive.

## Conclusion

The procedures established at the Atominstitut for sampling, irradiation, measurement and statistical analysis are optimized for analysis and grouping of archaeological ceramics. With low detection limits, small measurement errors and high reproducibility, all prerequisites for the successful analysis of ceramic artefacts are in place. The statistical evaluation of the data follows the procedures established in Bonn and produces reliable results (i.e. in D’Ercole et al. [31]). Comparability with international databases of ceramic analysis has been facilitated by expansion of the elemental spectrum measured but needs to be checked on an individual basis.
